# CD64 as a biomarker and therapeutic target in HAM/TSP and HTLV-1-associated Infective Dermatitis

**DOI:** 10.1186/1742-4690-8-S1-A95

**Published:** 2011-06-06

**Authors:** Ricardo Khouri, George Soares, Gilvaneia Silva-Santos, Giselia Santana, Theo Thepen, Lourdes Farre, Achilea Bittencourt, Giovanni Lopez, Michael Talledo, Eduardo Gotuzzo, Anne-Mieke Vandamme, Bernardo Galvao-Castro, Johan Van Weyenbergh

**Affiliations:** 1 Rega Institute, K.U. Leuven, Leuven, Belgium; 2 CPqGM-FIOCRUZ, Salvador-Bahia, Brazil; 3 Fraunhofer Institute, Aachen, Germany; 4 HUPES-UFBA, Salvador-Bahia, Brazil; 5 Tropical Medicine Research Center, UCH, Lima, Peru

## 

Previous studies have linked CD8+ T cell subset IFN-gamma production to HAM/TSP disease evolution [1,2], as well as its dependence on infected monocytes (Enose-Akahata et al. 2008). In addition, infective dermatitis (ID) can trigger HAM/TSP in children and adolescents [3]. Therefore, we quantified ex vivo expression of IFN-gamma/STAT1-regulated genes CD64 and CD95 in monocytes and neutrophils by flow cytometry in 44 subjects from Brazil and Peru, ranging from normal donors (ND), asymptomatics (AS), infective dermatitis (ID) and HAM/TSP to HAM/TSP+ID co-morbidity. Ex vivo CD64 expression in neutrophils was significantly associated with disease progression, with ND.
